# Lengthening and deformity correction about the knee using a magnetic internal lengthening nail

**DOI:** 10.1051/sicotj/2017014

**Published:** 2017-03-22

**Authors:** Austin T. Fragomen, S. Robert Rozbruch

**Affiliations:** 1 Limb Lengthening and Complex Reconstruction Service, Hospital for Special Surgery, Weill Medical College of Cornell University New York NY 10021 USA

**Keywords:** Internal lengthening nail, Limb deformity, Limb lengthening, Distraction, Malalignment

## Abstract

*Introduction*: The introduction of the internal lengthening nail (ILN) has changed the treatment of complex malalignment and shortening about the knee. Acute correction of the deformity and gradual lengthening through this osteotomy site has greatly simplified postoperative recovery. This manuscript is a review of the techniques that are currently being used in surgery.

*Methods*: The article is broken into two sections: distal femur osteotomy and tibia osteotomy. Each is addressed separately since they have different personalities. Also included are topics of particular interest that surface in ongoing conferences regarding the ILN. This work is a mix of expert opinion and best practice supported by peer reviewed publications on the topic.

*Results*: Most published series demonstrate excellent results with the ILN. Certain precautions are reiterated including avoiding mechanical failure, need for a percutaneous osteotomy, need for over-reaming, and the need for blocking screws.

*Discussion*: Current controversies will be brought to light and discussed. The reader should find this aspect particularly helpful in navigating this rapidly evolving field.

## Introduction

The introduction of the internal lengthening nail (ILN) has revolutionized how deformity surgeons approach the treatment of complex malalignment and shortening about the knee. Where once external fixation was required to manage simultaneous deformity with lengthening, internal fixation has utilized the tactic of acutely correcting the malalignment and gradually lengthening through this osteotomy site [1]. Some studies have even shown that the regenerate heals faster (a lower consolidation index) with ILN when compared with external fixation [2, 3]. Certain technical considerations are important to the success of this newer approach including well-placed blocking screws and the intraoperative use of external fixation to control the bony reduction during reaming. This review article aims to present successful strategies for deformity correction and lengthening through the distal femur and proximal tibia.

## Methods: femur

### Retrograde femoral nailing: surgical technique

The retrograde approach to the femur offers excellent control over distal femoral deformity. The distal femur is an ideal location for osteotomy due to its reliable bone formation during distraction osteogenesis. The challenge of the distal femur lies in its width which allows for the reamer and intramedullary (IM) nail to move in many possible directions. In order to secure a path for the nail to pass, blocking screws have been implemented. Well-placed blocking screws ensure that the reamer and IM nail will head in the proper direction both correcting the acute deformity and preventing lengthening-induced deformity. This concept of lengthening-induced deformity is well known to deformity surgeons with experience using external fixators but may not be so obvious to other orthopedic surgeons attempting deformity correction and lengthening with the IM nail. For retrograde nailing with a distal femoral osteotomy the distal bone fragment can be expected to flex during lengthening. This requires that a blocking screw be placed posterior to the nail at the time of the index surgery in anticipation of the impending deformity [3]. The mechanical axis will deviate laterally during lengthening at an expected quantity of 1 mm of translation per 1 cm of lengthening [4]. This circumstance is of particular relevance for lengthenings of 8 cm where 8 mm of lateral translation can occur. This scenario is less common in the setting of periarticular deformity correction, where average lengthening is 3 cm, and more common in the practice of stature lengthening, which is not the focus of this paper. With regard to stature lengthening, where an antegrade approach is typically used, an interesting phenomenon occurs: the nail and femur tend to bend into a few degrees of varus. This varus deflection counteracts the lateralization of the mechanical axis. Therefore, in more aggressive lengthenings, the use of an antegrade technique will typically keep the mechanical axis neutral. For a retrograde lengthening, the mechanical axis would need to be shifted the appropriate number of millimeters medially during planning to anticipate the valgus deviation. This will add a small amount of varus correction acutely to the correction. The reverse planning technique can be used as well [5].

### Location of the deformity

**Figure 1.**
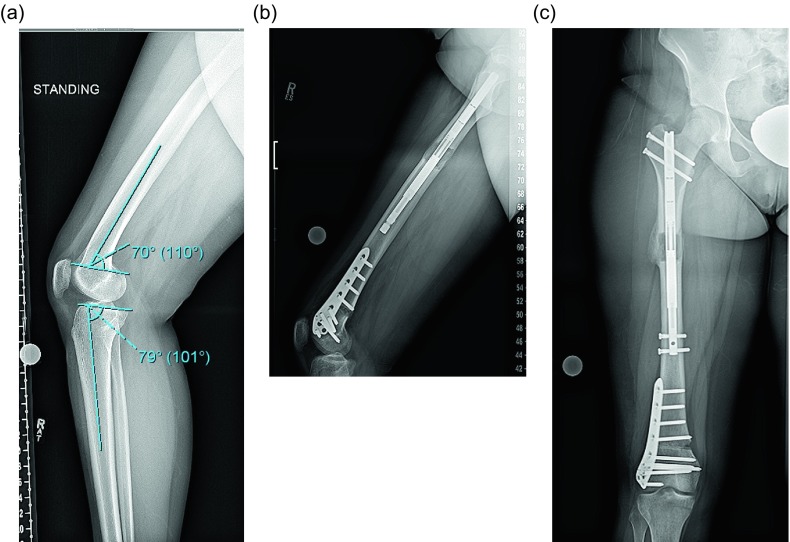
(a) This preop lateral radiograph shows a 13° flexion deformity of the distal femur. (b) Postop lateral shows a correction of the apex anterior deformity through an posterior opening wedge osteotomy stabilized with a plate and a proximal femoral lengthening with an ILN. (c) This far distal osteotomy would be difficult to control with an intramedullary implant.

The location of the deformity in the distal femur is important to determine. This technique is best suited for meta-diaphyseal deformities [6, 7]. A far distal deformity requiring a very “low” osteotomy may be better treated with a plate. An ILN can be inserted proximally to address rotation and shortening (Figures 1a–1c).

Surgical steps:

1. Preoperative planning: There are many ways to plan for the deformity correction and lengthening [5, 8]. Whatever technique is used will require two considerations: first, the existing deformity needs to be measured. Second, the expected lateral deviation of the mechanical axis that occurs during lengthening [4] needs to be considered. Then an osteotomy site is picked, translation is accounted for, blocking screw position is planned, and nail length and width are selected (Figures 2a–2c) [8].
  **Figure 2.**
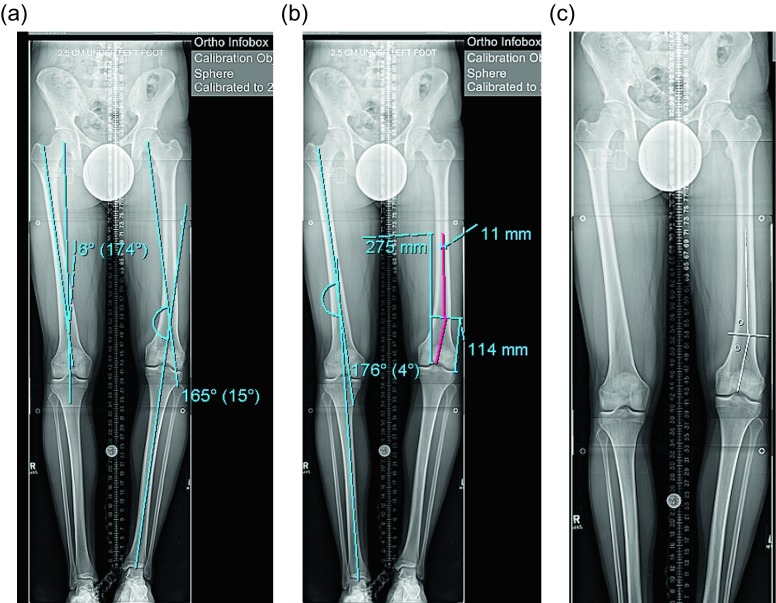
(a) This 51 inch AP radiograph allows for deformity planning with correction of varus and shortening. The mechanical axis planning is used to determine the magnitude and location of the deformity. (b) The red lines show the planned path of the ILN that will ensure correction of mechanical axis. The lines also show the length of the nail. (c) The white lines are placed to designate the path of the nail, and the circles are used to mark the location of the blocking screws. These radiographic plans are brought into the OR for comparison with intraop fluoroscopy shots.
2. Osteotomy site: The osteotomy site that was selected on the preoperative plan is located on the femur under fluoroscopy. A 10 mm incision is made in the lateral thigh at this level and taken through the iliotibial (IT) band. The incision can be extended for an IT band release. We release the IT band routinely for correction of valgus and lengthening and for large lengthenings (over 4 cm). A drill is then inserted with a protective sleeve, and multiple drill holes are created in one plane. This creates vent holes during reaming and marks the osteotomy site to help with blocking screw placement.
3. Blocking screws: When correcting coronal plane deformity blocking screws are inserted. The position of the screw needs to be decided as a part of the preoperative planning and replicated in the OR. For valgus deformity a blocking screw is placed lateral to the proposed path of the ILN (Figure 3a). For varus deformity the blocking screw is placed medial to the path of the nail (Figure 3b). An additional screw can be placed in the proximal fragment as well for optimal control. A blocking screw needs to be placed posterior to the path of the ILN (Figures 3c and 3d).
  **Figure 3.**
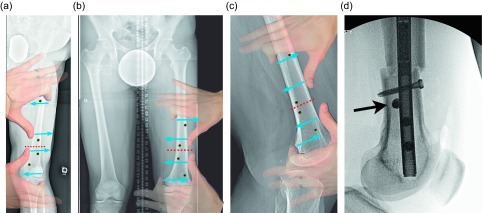
(a) This illustration shows the suggested position of the blocking screws for correction of a valgus deformity. The screws centered around the osteotomy are more typically used leaving the peripheral screws as an option. (b) The same planning can be used for correction of a varus deformity. The overlay of the hands is showing a principle called the “reverse rule of thumbs” whereby the bone is grabbed with the thumb and index fingers of both hands and a correction simulated. The blocking screws should be placed opposite the location of the thumb and index fingers. (c) In the sagittal plane, the blocking screws are inserted posterior to the ILN at the osteotomy site. The distal screw is the most important, but the proximal screw can also be used for a shorter nail. The peripheral screws are seldom needed. (d). This fluoroscopy shot shows ideal posterior blocking screw placement: close to the osteotomy site and lying against the ILN (arrow).
4. Half pins: A half pin is inserted into the distal femur using cannulated technique. A K-wire is inserted posterior to the path of the IM nail and checked using C-arm. The wire is then over-drilled with a cannulated drill bit through a small incision. The solid half pin (5–6 mm) is then inserted (Figure 4). A second half pin is inserted in the proximal femur, proximal to the tip of the ILN. Rotation should be accounted for in this step for later correction.
  **Figure 4.**
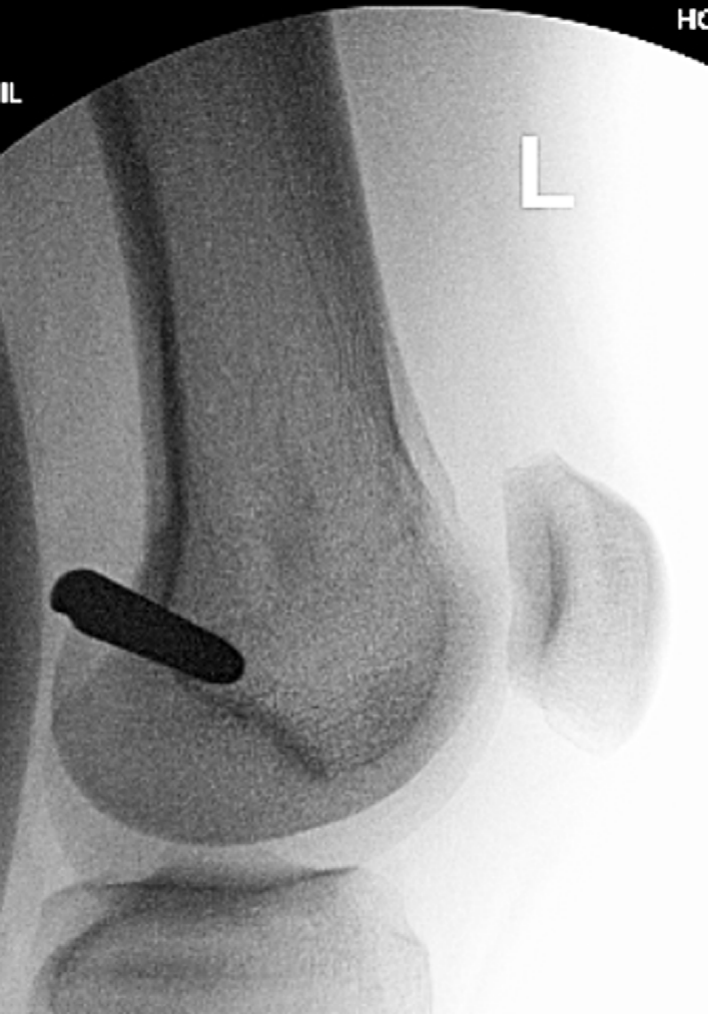
A 6 mm half pin is seen in the typical location posterior to the path of the ILN.
5. Distal fragment: The knee is flexed, and the distal femur is prepared through an arthrotomy either through or medial to the patellar tendon. A 2.4 mm K-wire is drilled in the center of the notch under AP and lateral fluoroscopy until it reaches the osteotomy site. The ACL 12 mm reamer is then inserted over the guide wire up to the osteotomy site taking care not to damage the patellar cartilage.
6. Osteotomy: The osteotomy is then performed with an osteotome through the previous drill hole incision under fluoroscopy. The reduction is performed with translation and rotational correction. The external fixator can then be placed to hold this reduction (Figure 5).
  **Figure 5.**
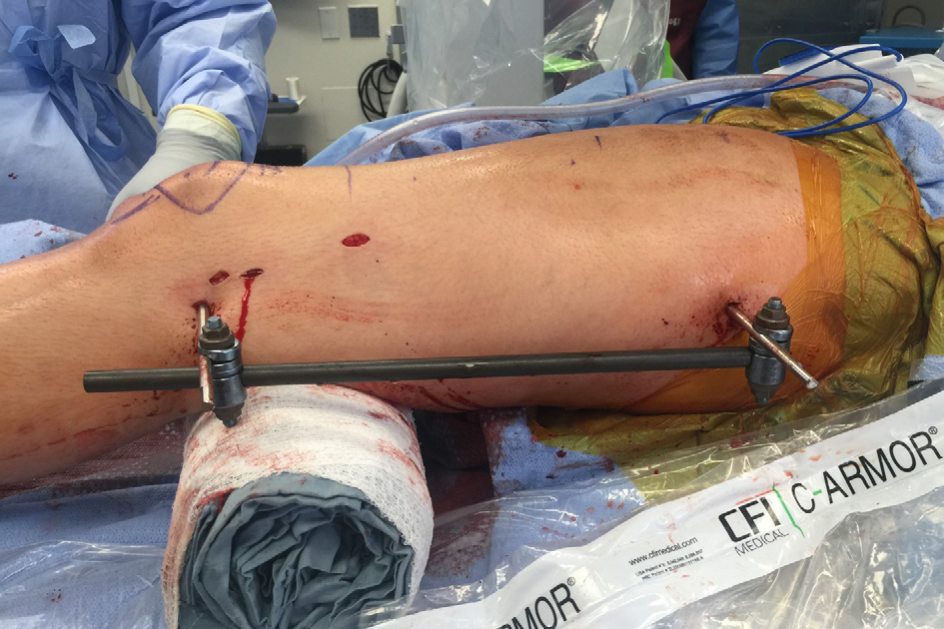
A simple pin-to-bar frame is used to hold the reduction of the osteotomy.
7. Reaming: Sequential reaming is then performed 1.5–2 mm over the nail diameter. There are different teachings on the correct way to prepare the canal. This will be further discussed.
8. ILN insertion: The external fixator is removed. The nail should slide into the canal without too much resistance. A mallet can be used, but if aggressive tapping is needed then we suggest removing the nail and reaming an additional 0.5–1.0 mm. The distal locking is then performed with the targeting device (Figure 6). The rotation and alignment are checked and adjusted with the half pins. The proximal screws are inserted with a free hand technique.
  **Figure 6.**
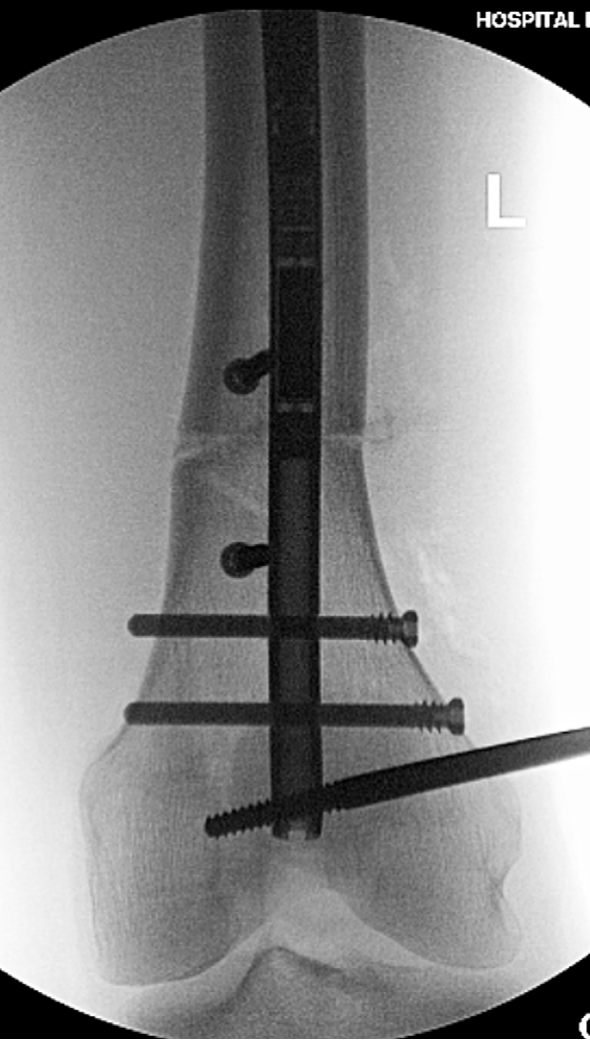
The AP fluoroscopy shot shows the distal femur after successful distal interlocking with the varus deformity corrected. The peri-osteotomy blocking screws are positioned to prevent varus deviation during lengthening. The external half pin is also seen in the field.
9. Follow-up: Lengthening begins after a latency period and proceeds at approximately 1 mm per day. The patient is followed at regular intervals with radiographs checking for regenerate quality and joint contractures (Figures 7a and 7b).
  **Figure 7.**
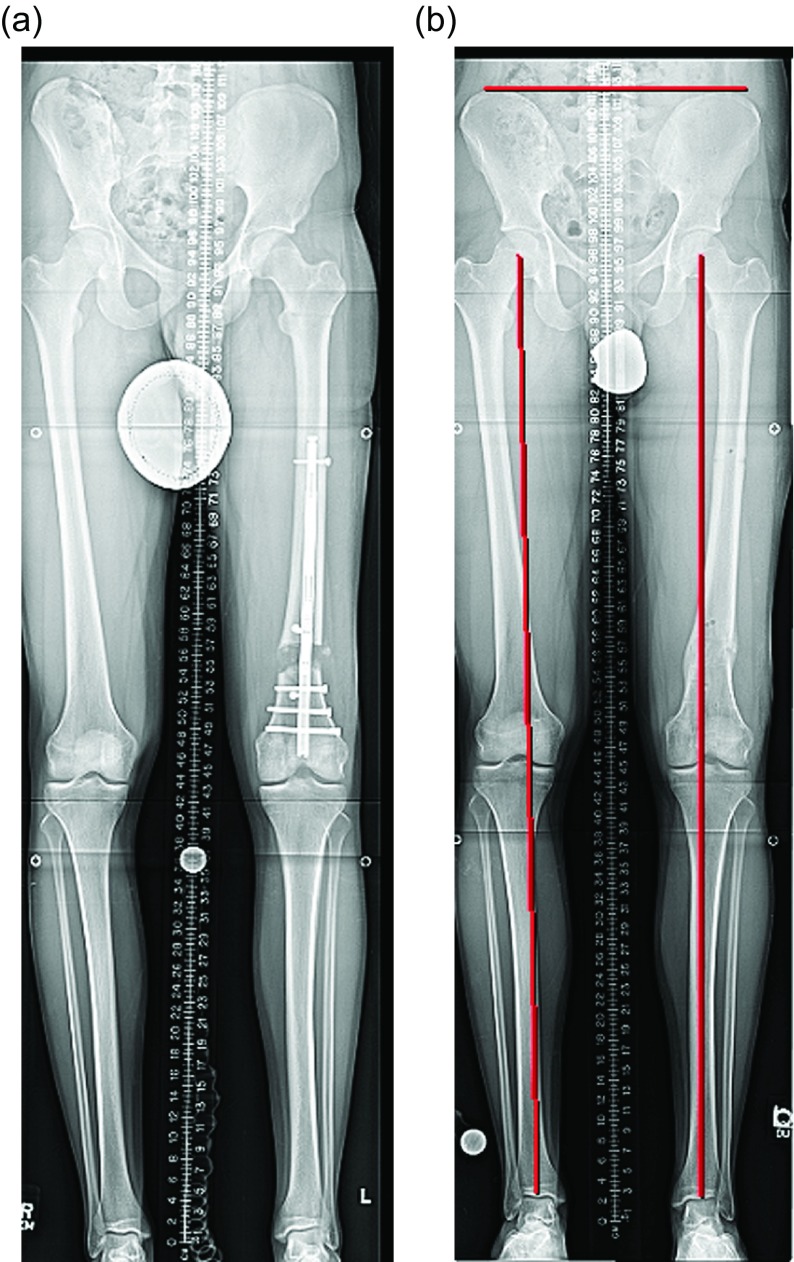
(a) The lengthening is complete and the limb length is checked. The regenerate bone is filling in but is still plastic enough to allow for fine-tuning of the length. The long-standing X-ray can be affected by hip and knee joint muscle contractures yielding a false length measurement. This needs to be considered before over-lengthening the limb. (b) The final alignment and length are checked after ILN removal.

## Results: femur

### Effect of acute deformity correction on the growth of the regenerate

An acute correction of the femur coronal plane deformity (average 7°, maximum 15–20°) will not impact the ability of regenerate bone to form through a distal femoral osteotomy [2, 3, 6, 7, 9–11]. At the 2016 Limb Lengthening and Reconstruction Society (LLRS) an abstract was presented reviewing a series of 27 patients all treated with simultaneous distal femoral deformity correction and lengthening with an ILN. In this cohort there were no nonunions after an acute correction of an average of 8° of deformity and gradual lengthening of an average of 3 cm of lengthening [12].

### Rate of lengthening

As a general rule G.A. Ilizarov taught the world that bony distraction achieved optimal osteogenesis when performed at a rate of 1 mm per day split into at least four separate adjustments of 0.25 mm per day. With the precision interlocking (IL) nails available, rate can be strictly controlled. In our practice, distraction needs to be started four days after surgery at the accelerated rate of 1.32 mm per day (0.33 mm four times daily) for four days and then slowed to a rate of 0.99 mm per day (0.33 mm three times daily). Rate is then further fine-tuned based on regenerate formation observed during the postop period. We have not experienced any implant-related premature consolidation or nonunion in the femur using this formula (one premature consolidation occurred due to a non-compliant patient) [13]. Singh et al. [14] reported excellent results starting lengthening post operative day (POD) 3 at 1 mm/day. Other authors have used a more classic rate of 1 mm per day following seven days of latency, however, in their series the external magnet was unresponsive and they had difficulty achieving the final goal and had premature consolidation. These problems may have been related to excess “drag” at the distraction site created by too slow a lengthening rate [15].

### Minimally invasive osteotomy

**Figure 8.**
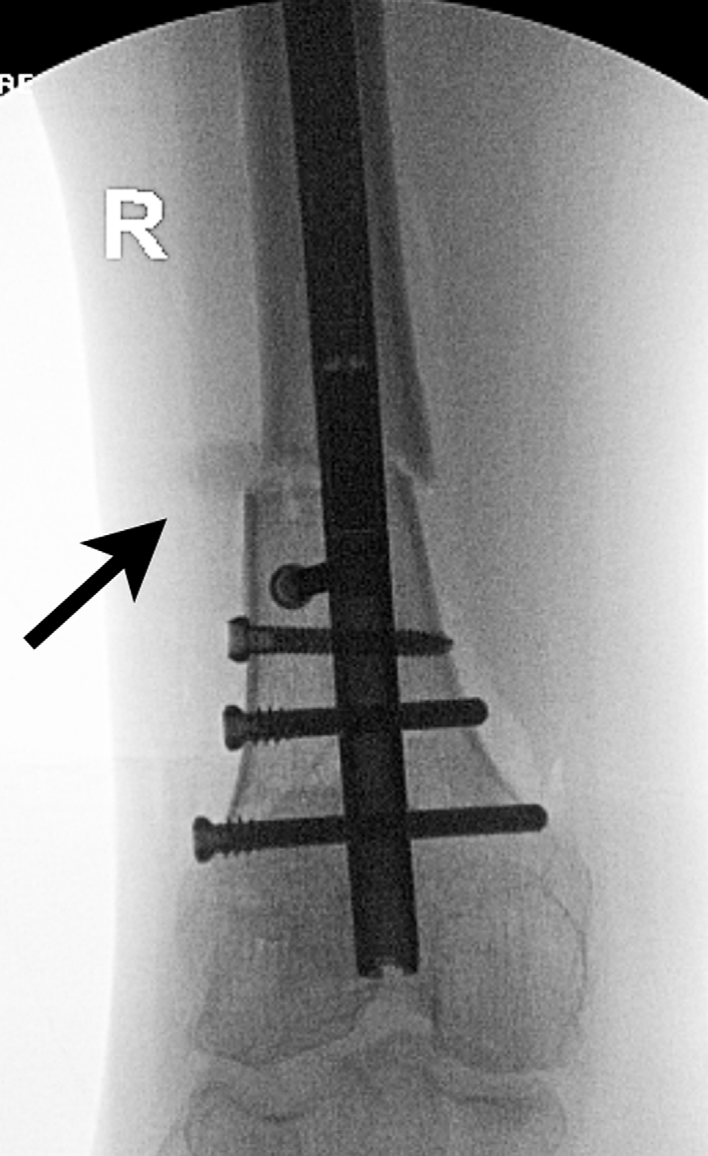
This intraop fluoroscopy frame shows a radio-opaque blush at the lateral aspect of the osteotomy site (arrow).

The role of minimally invasive osteotomy is believed to be paramount to the success of acute deformity correction followed by distraction. The creation of drill holes at the osteotomy site followed by reaming leads to the deposition of endosteal autograft at the osteotomy site. This pre-grafting is noticeable on the intraop radiographs (Figure 8). Osteotomy is then achieved without ever visualizing the bone. The osteotome is directed by feel and by fluoroscopy. It is possible that the success of acute correction is in part due to the minimally invasive nature of this osteotomy [6, 11–13, 15–17]. An open osteotomy may drain the site of the autograft and ruin the regeneration potential. Therefore, the excellent results achieved with this combination of techniques cannot be assigned to any one variable and deserve further study to determine the impact of each variable on the end product. One series reported 4/10 nonunions which may be due to a distraction rhythm of 0.5 mm BID or due to use of the reamer-irrigator-aspirator or due to a more open osteotomy technique [18].

### Pain control

The United States is exiting an era of heavy narcotic prescribing by launching an initiative to drastically cut down on opioids. The field of limb deformity surgery in the US has relied greatly on prolonged narcotic use for the duration of the lengthening process and often the entire time that external fixator is on the patient. Based on promising results in pediatric patients [19], adult surgeons are moving toward the aggressive use of acetaminophen and non-steroidal anti-inflammatories (NSAIDs) with minimal narcotic backup [20]. There is concern that the use of NSAIDs may slow the healing requiring a different rate of distraction. Alternatively, the use of narcotics may have negatively affected the regenerate formation [21] and abandoning them could positively affect the bone formation going forward. The results of this paradigm shift will be seen in the coming months.

### Intraoperative external fixation

The use of external fixation during this surgery is helpful for maintaining the deformity correction during the reaming process. Since the ILN will follow the path of the reamer we believe it is best to create that path with the bony deformity reduced and stabilized. The half pins also offer rigid control of the bone fragments. This helps to rotate the osteotomy to ensure it is complete, translate the bone fragments to achieve the desired reduction, and control the final rotation prior to interlocking. Some surgeons feel that the half pins and the external fixator are not necessary [6], however, they ensure a smooth procedure with reliable results [17]. One study showed that larger half pin size was associated with a more accurate correction of the mechanical axis [12].

### Rigid vs. flexible reaming

The decision of whether to ream the femoral canal using rigid reamers or flexible reamers has been debated extensively. The advantage of rigid reaming is that the sagittal curve of the femur is minimized allowing for less over-reaming of the canal. This in turn gives the ILN a tighter fit with less potential for malalignment at the time of insertion or during the lengthening process [6, 11]. If performed correctly the rigid reamer can preferentially remove posterior cortical bone which some surgeons feel is important [22]. There are several advantages to flexible reaming. It is more convenient since it does not require special reamers. Rigid reamers have the potential to notch the anterior cortex putting the femur at greater risk for fracture [16], a problem unlikely to happen with flexible reamers. Over-reaming with flexible reamers has not been a problem with excellent alignment and healing obtained [13, 17].

### Distal curved vs. straight ILN

**Figure 9.**
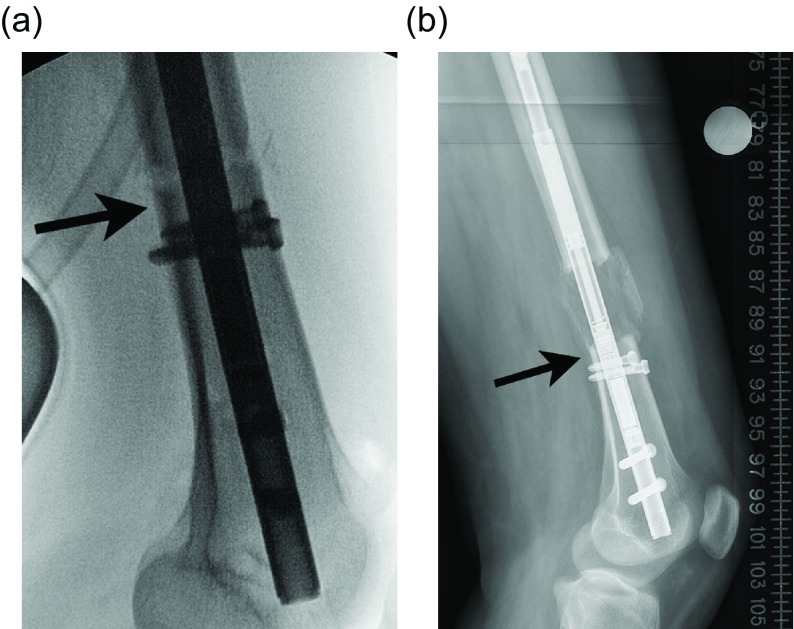
(a) The intraop fluoroscopy image shows contact of the posterior cortex of the distal fragment with the ILN at the osteotomy site (arrow). (b) After 5 cm of lengthening the distal fragment has not been able to flex due to the cortical abutment (arrow).

The ILN comes with both curved and straight distal options. There has been little discussion as to the advantages of each. There may be an increased tendency to create a procurvatum deformity when using the curved nail [22]. In cases where the surgeon wants to correct a procurvatum deformity at the osteotomy site, a curved nail can be rotated 180° to extend the osteotomy site. We have found that the straight entry ILN will often contact the posterior cortex obviating the need for a blocking screw (Figures 9a and 9b).

## Methods: tibia

### Tibial nailing: surgical technique

Acute deformity correction of the tibia followed by distraction with an ILN is gaining popularity. Tibial nailing is more challenging than retrograde femoral nailing with regard to alignment control. The entry point of the tibial nail is difficult to pinpoint due to the need to flex the knee with subsequent poor AP fluoroscopic imaging. The suprapatellar approach to tibial preparation may help with alignment precision [23] although this has never been studied in deformity patients. Proximal tibia osteotomy, much like proximal tibial fractures, often requires the use of blocking screws and external fixation. The posterior blocking screw is critical in preventing flexion of the osteotomy. Coronal plane blocking screws also ensure that the nail stays central on the AP view.

Surgical steps:

1. Preoperative planning: The tibia does not deviate from the mechanical axis during lengthening making planning straightforward. An osteotomy site is picked, translation is accounted for, blocking screw position is planned, and nail length and width are selected (Figures 10a and 10b). Nail width is often quite limited in the isthmus requiring either a thinner nail or a shorter nail.
  **Figure 10.**
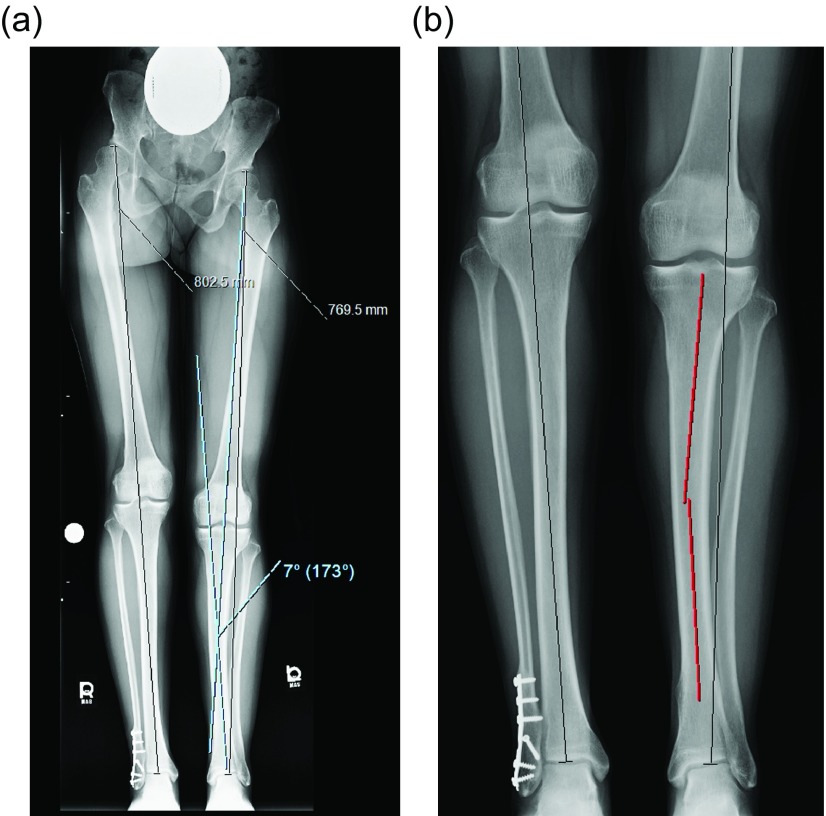
(a) The 51 inch standing film shows a diaphyseal valgus deformity and limb length discrepancy. (b) The path of the ILN is planned with the osteotomy site at the intersection of the red lines.
2. Osteotomy site: The osteotomy site that was selected on the preoperative plan is located on the tibia under fluoroscopy. A 10 mm incision is made in proximal leg. A drill is then inserted with a protective sleeve, and multiple drill holes are created in one plane. This creates vent holes during reaming and marks the osteotomy site to help with blocking screw placement.
3. Blocking screws: When correcting coronal plane deformity, blocking screws are inserted. The position of the screw needs to be decided as a part of the preoperative planning. The distance of the screws from the osteotomy site and cortex should be replicated in the OR. For valgus deformity a blocking screw is placed lateral to the proposed path of the ILN (Figure 11a). For varus deformity the blocking screw is placed medial to the path of the nail. An additional screw can be placed in the distal fragment as well for optimal control. The most critical blocking screw is placed posterior to the path of the IM nail in the proximal fragment. This will ensure the nail is not inserted in flexion and will prevent flexion from developing during lengthening (Figures 11b and 11c).
  **Figure 11.**
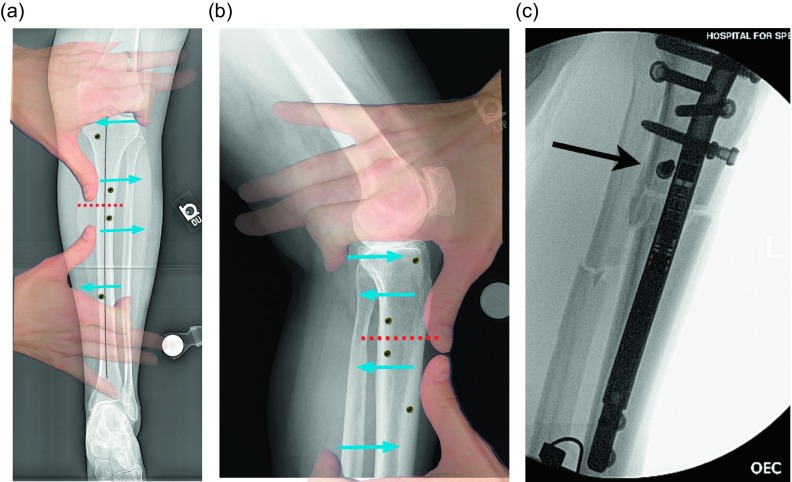
(a) This shows a different patient’s X-rays. The valgus correction is maintained by placing blocking screws in the concavity of the deformity (lateral aspect). Peripheral blocking screws can be used as well. (b) The sagittal plane requires at least one blocking screw in the proximal fragment near the osteotomy (the screw shown at the entry site of the ILN is theoretical and is never used). (c) This lateral film shows a well-placed posterior blocking screw (arrow).
4. Half pins: A half pin is inserted into the proximal tibia using cannulated technique. A K-wire is inserted posterior to the path of the IM nail usually superior and anterior to the fibular head and checked using C-arm. The wire is then over-drilled with a cannulated drill bit through a small incision. The solid half pin (5–6 mm) is then inserted. A second half pin is inserted in the distal tibia, distal to the tip of the ILN. Rotation should be accounted for in this step for later correction (Figure 12).
  **Figure 12.**
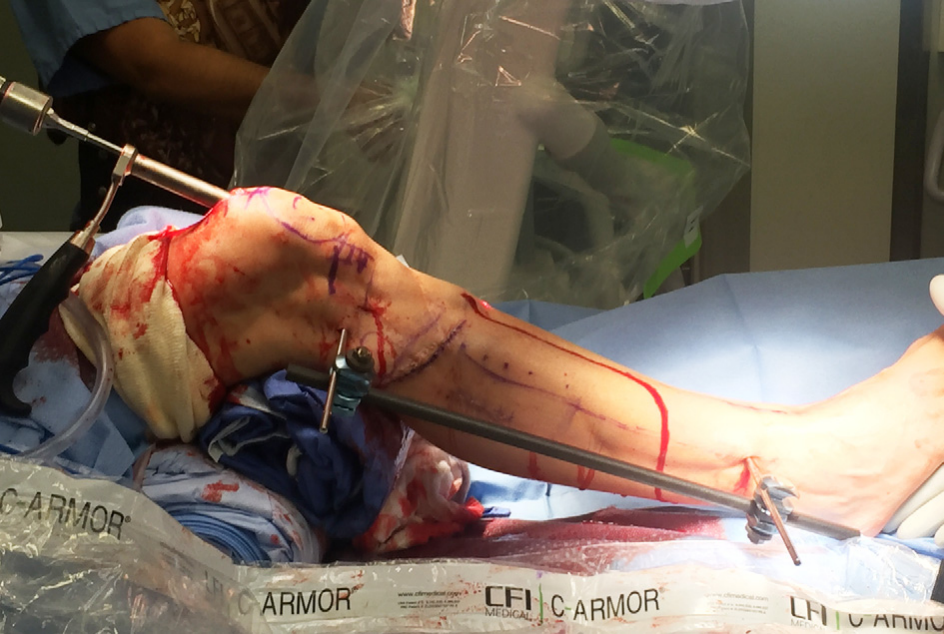
In this unrelated case, the external fixator helps to maintain the reduction of the deformity correction after osteotomy during reaming. A suprapatellar approach was used in this case.
5. Distal syndesmotic screw: The distal syndesmotic screw is placed to protect the ankle during distraction. We recommend a 4.5 mm fully threaded solid cortical screw.
6. Fibular osteotomy: The fibula is cut percutaneously by pre-drilling with a K-wire and then using an osteotome (Figure 13).
  **Figure 13.**
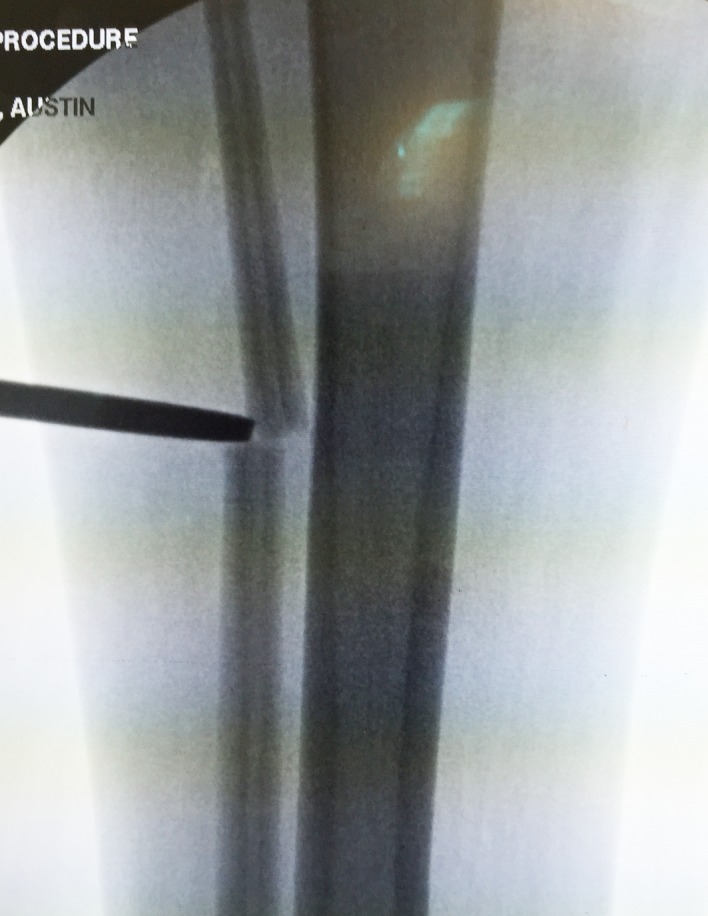
The osteotome is inserted through a limited incision under fluoroscopy.
7. Proximal fragment: The knee is flexed, and the proximal tibia is prepared through an arthrotomy either through or near the patellar tendon. A suprapatellar approach can also be used. A 2.4 mm K-wire is drilled under AP and Lateral fluoroscopy until it reaches the osteotomy site. The ACL 12 mm reamer is then inserted over the guide wire up to the osteotomy site.
8. Osteoplasty: The osteotomy is then performed with an osteotome through the previous drill hole incision under fluoroscopy. The reduction is performed with translation and rotational correction. The external fixator can then be secured to hold this reduction.
9. Reaming: Sequential reaming is then performed over-reaming the canal by 1.5–2 mm.
10. ILN insertion. The external fixator is loosened. The nail should slide into the canal without too much resistance. A mallet can be used, but if aggressive tapping is needed then we suggest removing the nail and reaming an additional 0.5–1.0 mm. The proximal locking is then performed with the targeting device. The rotation and alignment are checked and adjusted with the half pins. The distal interlock screws are inserted with a free hand technique.
11. Proximal syndesmotic screw: The proximal screw is placed using a cannulated technique. A guide wire is placed through the fibular head from lateral to medial. It is over-drilled and then a solid screw is inserted to prevent migration of the fibula during lengthening (Figure 14).
  **Figure 14.**
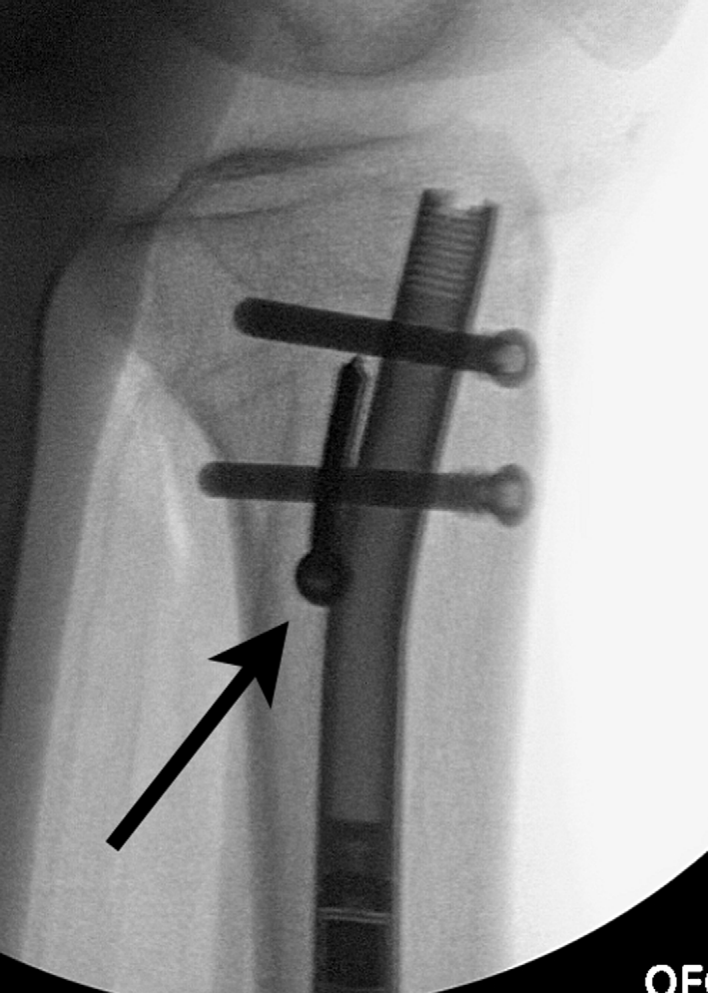
In this unrelated case, the proximal syndesmotic screw also served as the posterior blocking screw (arrow).
12. Fasciotomy: It is routine to perform a percutaneous fasciotomy of the anterior compartment when acutely correcting a tibial deformity to prevent compartment syndrome [18].
13. Follow-up: Lengthening begins after a latency period and proceeds at approximately 0.75 mm per day. The patient is followed at regular intervals with radiographs checking for regenerate quality and joint contractures (Figures 15a and 15b).
  **Figure 15.**
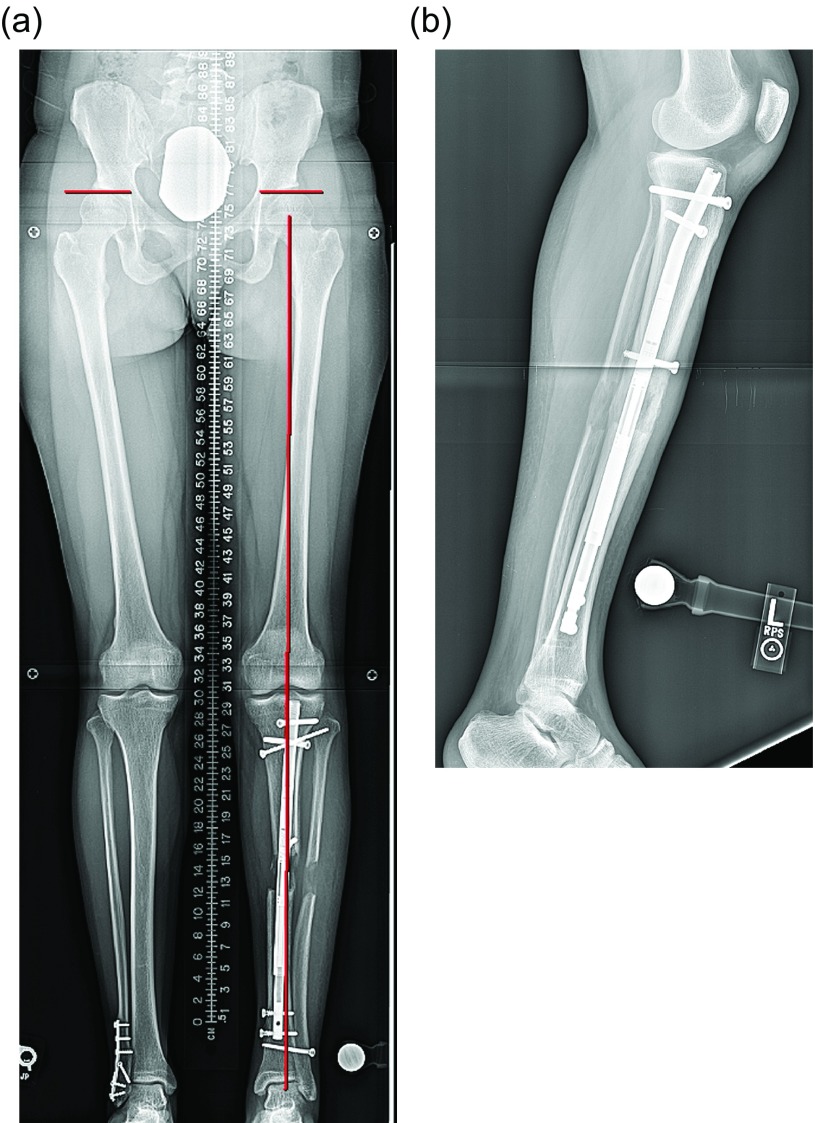
(a) The X-rays shown in Figures 15a and 15b are a continuation of the patient presented in Figures 10a and 10b. The end of distraction long-standing radiograph shows equal limb length and ideal alignment. (b) A lateral radiograph shows interval healing of the regenerate and ideal sagittal alignment.

## Results: tibia

### Prophylactic peroneal nerve decompression

It is clear that tibial lengthening places tension on the common peroneal nerve and can cause nerve damage [24, 25]. Special consideration is given to the correction of valgus or rotational deformities in conjunction with lengthening. These realignment procedures will further stretch the peroneal nerve and therefore, require a peroneal nerve release. This is done at the beginning of the surgery under tourniquet. For varus deformity correction with lengthening the peroneal nerve release is not standard. For larger lengthening goals (stature lengthening or greater than 27% increase in tibial length) the release should be strongly considered [26]. In all cases of tibia lengthening the common peroneal nerve is at risk for injury, and nerve function needs to be closely monitored postoperatively during the lengthening process [27].

### Will acute deformity correction affect the growth of the regenerate?

This topic is not as well studied as in the femoral counterpart. Acute deformity correction of in the tibia may affect the quality of the regenerate, as measured by the consolidation index and circumferential healing, greater than the effect seen in the femur. The consolidation index (CI) for femoral lengthening has been excellent, averaging 24–32 days/cm [9, 11, 14, 16] (faster than that seen with external fixation lengthening). These studies include very few tibia cases obscuring our ability to compare CI among tibia lengthening cases. The protocol we use for the tibial lengthening is slower than for femoral lengthening. The latency is seven days after which the tibia is lengthened at a rate of 1 mm per day split into four 0.25 mm lengthening sessions. This is done for four days and then slowed to 0.25 mm three times per day. This slower lengthening schedule has been helpful in protecting the osteotomy site’s ability to heal.

### Tibial bone healing

The tibial consolidation rate is significantly slower than that of the femur using ILN [11]. The situation is further complicated by the asymmetric healing of the different tibial cortices. The deficiency is typically in the anterior portion of the regenerate. Responding to the deficient regenerate is complicated since a slower rate of distraction will help the anterior cortex while placing the posterior cortex at risk for premature consolidation. Most surgeons will avoid consolidation in favor of the posterior cortical healing. Surgical treatment is varied depending on the severity of the anterior defect but can include observation, percutaneous drilling, and injection of bone marrow aspirate concentrate, exchange nailing, open bone grafting, or a combination.

## Discussion

The ILN is a great advancement in the area of limb deformity and lengthening, particularly in the femur. Surgeons are still learning the capabilities and shortcomings of these devices. The quality of the available implants continues to improve. The tibia continues to challenge surgeons’ abilities to control deformity and bone formation during lengthening and remains an area where external fixation is incredibly useful.

## Conflict of interest

Author ATF received no funding or financial benefit related to this work. Author ATF does function as a consultant for NuVasive, Smith & Nephew, and Synthes. Author SRR received no funding or financial benefit related to this work. Author SRR does function as a consultant for NuVasive, Smith & Nephew, and Stryker.
